# A fatal neonatal case of fungemia due to *Exophiala dermatitidis*—case report and literature review

**DOI:** 10.1186/s12887-022-03518-5

**Published:** 2022-08-10

**Authors:** Alexandra Mpakosi, Maria Siopi, Maria Demetriou, Vasiliki Falaina, Martha Theodoraki, Joseph Meletiadis

**Affiliations:** 1 Department of Microbiology, General Hospital of Nikaia Agios Panteleimon, Athens, Greece; 2 Clinical Microbiology Laboratory, Attikon University Hospital, Medical School, National and Kapodistrian University of Athens, 1 Rimini str, Haidari 124 62, Athens, Greece; 3 Department of Microbiology, Metaxa Memorial Anticancer Hospital of Piraeus, Piraeus, Greece; 4 Neonatal Intensive Care Unit, General Hospital of Nikaia Agios Panteleimon, Athens, Greece

**Keywords:** *Exophiala dermatitidis*, Premature neonate, Extremely low birth weight, Fungemia, Black yeast-like fungus

## Abstract

**Background:**

Systemic infections caused by the black yeast-like fungus *Exophiala dermatitidis* are rare, but are associated with high mortality especially in immunocompromised patients. We report the first case of *E. dermatitidis* fungemia in a premature extremely low birth weight (ELBW) neonate who succumbed despite antifungal therapy with liposomal amphotericin (AMB) and fluconazole. A systematic review of all fungemia cases due to *E. dermatitidis* was also conducted aiming for a better understanding of the risk factors, treatment strategies and outcomes.

**Case presentation:**

A male, ELBW premature neonate, soon after his birth, developed bradycardia, apnoea and ultimately necrotizing enterocolitis with intestinal perforation requiring surgical intervention. Meanwhile, he had also multiple risk factors for developing bloodstream infection, such as intubation, mechanical ventilation, central venous catheter (CVC), parenteral nutrition, empirical and prolonged antibiotic use. His blood cultures were positive, firstly for *Acinetobacter junii* and then for *Klebsiella pneumoniae* together with *E. dermatitidis* while on fluconazole prophylaxis and antibiotic empiric therapy. Despite the treatment with broad spectrum antibiotics, liposomal AMB and fluconazole, the newborn succumbed. A literature review identified another 12 *E. dermatitidis* bloodstream infections, mainly in patients with hematologic malignancies and solid organ transplant recipients (61%), with overall mortality 38% despite CVC removal and antifungal therapy.

**Conclusions:**

Due to the rarity of *E. dermatitidis* infections, little is known about the characteristics of this yeast, the identification methods and the optimal therapy. Identification by common biochemical tests was problematic requiring molecular identification. Resolution of neonatal fungemia is difficult despite proper antifungal therapy especially in cases with multiple and severe risk factors like the present one. Therapeutic intervention may include CVC removal and treatment for at least 3 weeks with an azole (itraconazole or fluconazole after susceptibility testing) or AMB monotherapy but not echinocandins or AMB plus azole combination therapy.

## Background

*Candida s*pecies remain the leading cause of invasive fungal infections (IFIs) in neonates. The mortality rate due to *Candida* sepsis is high (25–54%) reaching 70% in very low birth weight newborns. *C. albicans* has been historically the commonest bloodstream pathogen in neonates followed by *C. parapsilosis* [1]. However, an increasing frequency of less common yeasts as emerging causes of neonatal bloodstream infections has been recently reported challenging the current therapeutic approaches [2].

Infections caused by *Exophiala* spp. are typically seen in immunocompromised hosts and manifest most commonly as subcutaneous disease. Systemic infections are exceedingly rare and are associated with significant morbidity and mortality. The most prominent representative of the genus is *Exophiala dermatitidis*. Given that the diagnosis and therapy of *E. dermatitidis* infections represent a challenge, we aim to draw the attention to the detection and identification of this slow growing fungus that might lead to delayed/failed treatment. We herein report the first *E. dermatitidis* fungemia case in a premature extremely low birth weight (ELBW) neonate and we review the literature in order to identify optimal therapeutic strategies.

## Case presentation

A 27 weeks preterm male newborn, weighing 810 g, was born vaginally, following premature rupture of the membranes of the amniotic sac 16 h prior to delivery. His mother developed disseminated intravascular coagulation of unspecified cause, with vaginal bleeding and gingivitis and needed plasma and platelet (PLT) transfusions. She had been treated with cortisone for her newborn’s pulmonary maturity 12 h before delivery. In the maternal medical history four previous missed abortions were reported, but her testing for thrombophilia was negative. In this case, she was treated with acetylsalicylic acid from the first trimester of pregnancy and with low molecular weight heparin during the last month of pregnancy because of fetal growth retardation. After birth, the newborn was intubated immediately because of bradycardia and apnoea with Apgar score 1 → 5 and 5 → 9. The umbilical cord pH was 6.9. Surfactant was administered once (200 mg/kg) and ampicillin (200 mg/kg/d) and gentamicin (5 mg/kg/48 h) were initiated empirically.

On day 5, he was admitted to the neonatal intensive care unit (NICU), where was extubated on NCPAP FiO_2_ 21%, PEEP 6 cm H_2_O, and besides the treatment above, fluconazole (FLC 3 mg/kg/d twice per week) was prophylactically added.

On day 7, he developed symptoms and signs of necrotizing enterocolitis (NEC) with abdominal distension, tenderness and intramural gas (pneumatosis intestinalis) in plain abdominal X-Ray. His laboratory results demonstrated white blood cells (WBCs) 12,000/mm^3^, PLTs 167,000/mm^3^, and C-reactive protein (CRP) 10 mg/L. The infant was re-intubated and the antibiotic therapy was modified to meropenem (40 mg/kg/24 h), amikacin (15 mg/kg/36 h) and metronidazole (7 mg/kg/48 h) and maintained for 10 days. In addition, the umbilical arterial catheter that had been inserted on the first day of life, was accordingly removed**.** However, intestinal perforation developed 24 h later and surgical intervention with resection of the necrotic bowel was required. At the same time an ileostomy was performed.

On day 11, a peripherally inserted central venous catheter (CVC, long-line) was placed that was maintained for a month.

On day 17, tachycardia occurred and his laboratory results showed increased WBCs (20,000/mm^3^ with 75% neutrophils), reduced thrombocytes (116,000/mm^3^) and increased CRP (max 99 mg/L). The blood culture was positive for *Acinetobacter junii*, susceptible, among other antibiotics, to meropenem and gentamicin. Six days after initiation of meropenem 40 mg/kg/24 h and gentamicin 4.5 mg/kg/36 h the blood cultures became negative. The treatment continued for another 2 weeks. Lumbar puncture culture was negative. The long-line culture was negative.

On day 42, a Hickmann CVC was implanted.

On day 44, his general condition was worsening again with apnoea, tachycardia, reduction of WBCs (2000/mm^3^) and PLTs (41,000/mm^3^) and increase of CRP (max 99 mg/L). Blood cultures that were collected on the same day, became positive for multidrug-resistant *Klebsiella pneumoniae* following 24 h of incubation. A treatment with colistin (75,000 IU/kg/d) and aztreonam (90 mg/kg/24 h) was initiated. His critical condition did not resolve and a black yeast identified as *E*. *dermatitidis* with low minimum inhibitory concentration (MIC) to amphotericin B (ΑΜΒ) as described below was detected on day 48, in combination with *K. pneumoniae* (coinfection), in blood cultures collected on day 45. Liposomal ΑΜΒ (7 mg/kg/24 h) and FLC (6 mg/kg/48 h) were added to treatment with antibiotics, but even 7 days after the blood cultures remained positive for the same microorganisms (5 blood cultures in 7 days). The Hickmann CVC was not removed because of the neonate’s critical situation due to multiple difficulties associated with prematurity, ELBW, NEC, parenteral nutrition, and administration of antibiotics. During these 7 days**,** the neonate’s general condition deteriorated, exhibiting haemorrhagic diathesis, free and encapsulated fluid in the peritoneal cavity, and echogenic appearance of the small intestine on ultrasound. The cerebral ultrasound showed unilateral hyperechogenicity of white matter and the thalamus. The abdominal ultrasound depicted swollen gallbladder and kidney hyperechogenicity. Ocular findings of pre-plus in Zona II (vascular abnormalities of the posterior pole) were observed in the ophthalmologic exam, while no signs of endophthalmitis were found. Urine cultures were negative for yeasts. Although the laboratory results were stable (WBCs 9800/mm^3^, PLTs 25,000/mm^3^, CRP 20 mg/L), the newborn developed mucosal bleeding in the next few days and apart from the conventional coagulation tests, the rotational thromboelastometry was used for the haemostatic derangement evaluation. Despite the transfusions with fresh frozen plasma and PLTs required, massive pulmonary haemorrhage occurred and the infant died at 55 days of life.

## Species identification and susceptibility profile

**Fig. 1:**
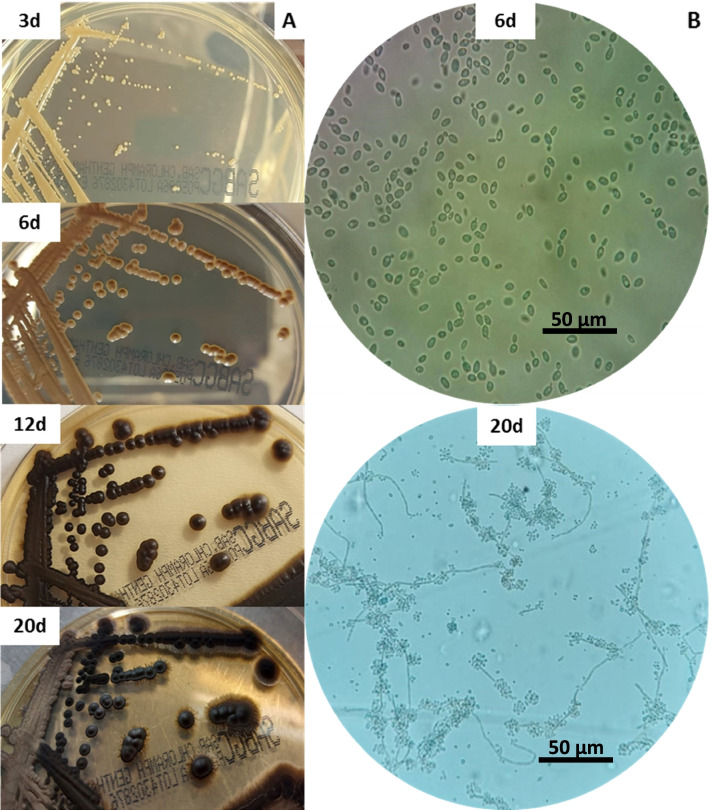
Macroscopic and microscopic characteristics of *Exophiala deramatitidis*. **A** Colonial morphology over time after incubation at 30 °C on Sabouraud dextrose agar with gentamicin and chloramphenicol. **B** Micromorphology over time showing the presence of yeasts cells and septate hyphae with conidial heads (original magnification 400x)

Positive blood cultures were inoculated onto blood agar, MacConkey agar and chocolate agar incubated at 37 °C, as well as Sabouraud dextrose agar with gentamicin and chloramphenicol (Oxoid) incubated at 30 °C. After 3 days of incubation, slow-growing pale brown colonies with smooth, glossy and yeast-like appearance were observed. Subsequently, colonies became suede-like developing an olivaceous-grey mycelium and a dark brown pigment was produced in the agar (Fig. 1A). Microscopy (Nikon Eclipse E200) revealed numerous ovoid to elliptical conidia (initial yeast-like phase), while with the development of the mycelium, septate hyphae with annellated zones with heads of globose to cylindrical conidia were produced (Fig. 1B). The fungus was identified as *Exophiala* spp. based on its colonial and microscopic features. Biochemical identification with the VITEK 2 Compact automated system (BioMeriéux, Marcy l’Etoile, France) yielded an unidentified organism result, while the numerical profile (70771 + 15) obtained by the Auxacolor identification system (Bio-Rad, Hercules, CA, USA) was not included in the interpretation table. Species identification was verified by sequencing the ITS1-5.8S-ITS2 region as previously described using ITS1 (5-TCCGTAGGTGAACCTGCGG-3) and ITS4 (5-TCCTCCGCTTATTGATATGC-3) primers [3]. High sequence alignment (> 99%) was found in Genbank Blast analysis with *E. dermatitidis* (GenBank Accession No MW885828).

*In vitro* antifungal susceptibility was determined using the commercially available colorimetric broth microdilution method Sensititre YeastOne (Thermo Fisher Scientific, Cleveland, OH) in accordance with the manufacturer’s recommendations after incubation at 37 °C for 48 h since the growth control well was only faintly purple after 24 h of incubation. The MIC values determined as the first blue well corresponding to complete growth inhibition (first purple well corresponding to partial growth inhibition is shown in parenthesis) were 0.25 mg/L for AMB, 4 (2) mg/L for FLC, 0.03 (0.015) mg/L for voriconazole (VRC), posaconazole (POS) and itraconazole (ITC), 4 (2) mg/L for flucytosine (5-FC), > 8 (8) mg/L for micafungin (MFG), > 8 (4) mg/L for anidulafungin (AFG) and 8 (4) mg/L for caspofungin (CAS). The isolate’s antifungal susceptibility profile was retrospectively tested following the EUCAST broth microdilution reference methodology [4]. The 48 h MICs (complete visual growth inhibition) were 0.25 mg/L for AMB, 16 mg/L for FLC, 0.125 mg/L for VRC and ITC, 0.5 mg/L for isavuconazole (ISA) and 0.06 mg/L for POS. Echinocandins’ minimal effective concentrations were 4 mg/L for both AFG and MFG and 2 mg/L for CAS. The isolate was stored (normal saline with 10% glycerol, -70 °C) in the culture collection of the Clinical Microbiology Laboratory’s Mycology Unit of “Attikon” University hospital (Athens, Greece) as AUH1807.

## Discussion and conclusion

Black yeasts, as well as their filamentous relatives, are responsible for a large number of serious and life-threatening infections in both immunocompetent and immunocompromised hosts. They may also occur as opportunistic pathogens of vertebrate hosts. Black yeasts reproduce by budding cells and their colonies are pale, gelatinous with dark brown or black colour, while in other related genera hyphal reproduction predominates [5]. However they are all able to form true hyphae. They usually cause localized infections, but haematogenous dissemination may also occur especially by two species of genus *Exophiala*, *E. dermatitidis* and *E. spinifera* [5]. Both can cause invasive, life-threatening infections with high mortality rates of up to 80% [6] due, probably, to the extracellular polysaccharide on yeast cells, through which they can escape from the host immunosurveillance processes [6].

*E. dermatitidis*, when disseminated, it mostly affects the central nervous system probably because of neutropism and the higher level of free iron than in serum [5, 7, 8]. Additionally, it colonises the respiratory tract of cystic fibrosis patients and the intestinal tract of immunocompromised hosts. It causes cutaneous infections such as otitis external, keratitis and onychomycosis in immunocompetent individuals [5, 6, 8]. *E. dermatitidis* is found in warm and moist environments as in steam bath, dishwashers, sauna facilities and creosoted railway sleepers [6, 8]. The melanised thick multi-layered cell wall is believed to be a major virulence factor leading to reduced susceptibility to antifungal drugs. In addition, by its dimorphic character it can survive by both morphological forms, yeast or hyphae, being also capable of biofilm formation which is a major cause of recurrent infection and leads to resistance to antifungals [8].

Immunity (innate and adaptive) is required for the response to black yeasts [5]. Several of the invasive infections were occurred in patiens with congenital imperfect defense system, including CARD9 deficient patients [6]. In the present study we describe a fatal fungemia case due to *E. dermatitidis* in a premature neonate. It is well known that premature neonates are at particularly increased risk of developing invasive infections with excessive case fatality due to their low birth weight, poor nutrition, enteral malabsorption, insufficient microbial defences, underdeveloped anatomic barriers and immune system. Major risk factors for fungemia include the presence of intravascular catheters, use of parenteral hyperalimentation and treatment with broad-spectrum antibiotics [1]. In our case prematurity (gestation age of 27 weeks), ELBW (810 g), respiratory distress syndrome (RDS), NEC, parenteral nutrition, mechanical ventilation, use of broad-spectrum antibiotics, surgical intervention with intestinal resection and intravascular catheters were the predisposing factors for fungemia. The *E. dermatitidis* infection occurred on day 45 when the neonate had already been in a critical state after the abdominal surgery and the implantation of the Hickmann CVC. Therefore, the degree of contribution of the *E. dermatitidis* infection to the death of the newborn is unclear. However, his state got worse after he acquired *E. dermatitidis* and multidrug-resistant *K. pneumoniae* infection. Simultaneous infection with more than one organism (concurrent fungemia and bacteremia) is not uncommon in very low birth weight infants [9]. After 7 days of antifungal and antibiotic treatment, the blood cultures remained positive for the same microorganisms and he died shortly after. Resolution of fungemia in neonates is slow despite proper antifungal treatment [1]. It is possible, but not certain, that bloodstream invasion occurred through the Hickmann CVC. Nevertheless, the presence of a CVC may be necessary for *E. dermatitidis* to establish bloodstream infection as the fungus finds the opportunity to adhere, produce extracellular matrix and grow as biofilm that helps the persistence of fungemia [10]. It is well known that the prognosis of any rare fungal infection is very poor and this type of infection is often a cause of high mortality although it is not always attributed to fungal infections [11].

An overview of all known 13 fungemia cases (12 previously published and our case) due to *E. dermatitidis* is shown in Table 1. According to these data, the median (range, interquartile range) age of patients was 47 (0.1–75, 55) years, 7/13 (54%) were male, 5/13 (38%) and 3/13 (23%) with hematologic and solid organ malignancies, respectively with 5/8 (62%) on chemotherapy, 5/13 with other underlying conditions (2 prematurity, 1 colectomy, 1 chronic obstructive pulmonary disease and cirrhosis, 1 HIV infection). In all cases a CVC was present. Overall mortality was 5/13 (38%). In 12/13 (92%) cases CVC was removed, whereas all patients received antifungal treatment: 2/13 (15%) AMB (1 survived, 1 died), 4/13 (31%) AMB and azole [1 VRC and 1 FLC (both died), 2 ITC as sequential (both survived)], 1/13 (8%) AMB and 5-FC (survived), 1/13 (8%) ITC (survived), 2/13 (15%) FLC (survived), 1/13 (8%) VRC after failure of combined therapy with MFG and liposomal AMB (survived) and 2 echinocandins monotherapy (both died). Azoles were given in 8/13 (62%) patients, 4/8 (50%) in combination with AMB (2 concomitantly with VRC/FLC who died and 2 sequentially with ITC who survived), 1/8 (12%) patient who survived after MFG and VRC treatment and then VRC orally and 3/8 (37%) as ITC/FLC monotherapy (all survived). Echinocandins were given in 5/13 (38%) patients (2 prophylaxis, 3 therapy) with 4/5 (80%) died. Prophylactic strategies are important for immunocompromised hosts during situations such as graft-versus-host disease (GVHD) (case 7), metastatic lung cancer with chemotherapy and parenteral nutrition (case 6), umbilical cord blood transplantation after graft failure (case 9), lymphoma (case 11) and ELBW (present case). Based on *in vitro* antifungal susceptibility testing, high MICs (> 1 mg/L) were found for FLC (100%), echinocandins (75–100%) and 5-FC (100%) compared to AMB, VRC, POS and ITC (0%) with VRC, POS and ITC MICs < 0.25 mg/L and AMB MICs < 1 mg/L.

**Table 1:** Overview of published fungemia cases due to *Exophiala dermatitidis* excluding outbreaks

Country, Year (reference)	Gender/ Age (years)	Predisposing factors	*In vitro* susceptibility (MICs (mg/L), testing method)	Treatment	Outcome
1. Germany, 1994 [12]	M/3.5	ALL Chemotherapy (totally implanted CVAD)	ND	CVC removal AMB and 5-FC (3 weeks)	Survival
2. Netherlands, 1994 [13]	M/5	ALL Chemotherapy (totally implanted CVAD)	ND	CVC removal ITC (8 weeks)	Survival
3. United States of America, 1995 [14]	M/3	HIV infection CVC for continuous zidovudine infusion	ND	CVC removal AMB (> 1 month) followed by ITC (5 months)	Survival
4. United Kingdom, 1995 [15]	F/53	Colectomy Long-term parenteral nutrition Prolonged antibiotic administration	ND	CVC removal FLC (4 days)	Survival
5. United States of America, 2002 [16]	F/61	Metastatic breast cancer Chemotherapy (totally implanted CVAD)	ND	CVC removal AMB (total dose 1260 mg) followed by ITC (8 weeks)	Survival
6. Taiwan, 2005 [17]	F/58	Metastatic lung cancer Chemotherapy (totally implanted CVAD) Parenteral nutrition	FLC 48 mg/L AMB 0.25 mg/L (NA)	Empirical coverage with FLC CVC removal AMB (17 days)	Death (*P. aeruginosa* septic shock, BC clearance after 4 days AMB treatment)
7. United States of America, 2014 [18]	M/57	Relapsed lymphoma HSCT GVHD PICC	FLC 8 mg/L ITC 0.25 mg/L VRC 0.125 mg/L POS 0.125 mg/L AMB 1 mg/L 5-FC > 64 mg/L CAS 8 mg/L AFG 8 mg/L MFG 8 mg/L TRB 0.06 mg/L (NA)	Prophylactic coverage with MFG CVC removal Switch to VRC and L-AMB (10 days)	Death (10 days after discontinuation of antifungal therapy due to transition to comfort care measures, BC clearance after 2 days of VRC and L-AMB)
8. Japan, 2012 [19]	F/47	Metastatic lingual and esophageal cancer Malnutrition Chemotherapy (totally implanted CVAD)	MFG > 16 mg/L (NA)	CVC removal MFG (12 days)	Death
9. Japan, 2017 [20]	M/45	Myelofibrosis AML UCBT Prolonged neutropenia	AMB 0.5 mg/L MCF > 16 mg/L FLC > 16 mg/L (CLSI M27-A3)	Prophylactic coverage with MFG CVC removal L-AMB (49 days)	Death (negative BC after 28 days treatment with L-AMB)
10. Argentina, 2019 [10]	M/75	Cirrhosis COPD CVC	AMB 0.125 mg/L CAS 0.008 mg/L AFG 0.008 mg/L (CLSI M38-A2)	CVC removal AFG (until death)	Death (negative BC after CVC removal and 5 days AFG treatment)
11. Japan, 2019 [21]	F/67	Follicular lymphoma recurrence PICC	5-FC 2 mg/L AMB 0.25 mg/L CAS 4 mg/L FLC 8 mg/L ITC 0.25 mg/L MFG 8 mg/L MCZ 0.5 mg/L VRC 0.06 mg/L (commercial ASTY® colorimetric panel)	CVC removal Empirical coverage with MFG (12 days) MFG and L-AMB (5 days) MFG and VRC (8 days) VRC (6 days)	Survival (positive BC after 12 days treatment with MFG, positive BC after MFG and L-AMB treatment, negative BC after MFG and VRC treatment)
12. India, 2020 [22]	F/0.2	Late preterm infant Mitochondriopathy and inborn error of metabolism Treatment with broad spectrum antibiotics CVC	AMB 1 mg/L FLC 4 mg/L VRC ≤ 0.03 mg/L ITC ≤ 0.03 mg/L POS 0.06 mg/L 5-FC 4 mg/L CAS 2 mg/L MFG 2 mg/L AFG 4 mg/L (CLSI M27-A3)	CVC removal FLC (21 days)	Survival
13. Present case (Greece, 2021)	M/0.1	Prematurity and ELBW (810 g) Intubation, mechanical ventilation, RDS Abdominal surgery, NEC, parenteral nutrition Treatment with broad spectrum antibiotics CVC	AMB 0.25/0.25 mg/L FLC 4/16 mg/L VRC 0.03/0.125 mg/L POS 0.03/0.06 mg/L ITC 0.03/0.125 mg/L ISA ND/0.5 mg/L 5-FC 4/ND mg/L MFG > 8/4 mg/L AFG > 8/4 mg/L CAS 8/2 mg/L (commercial Sensititre YeastOne® colorimetric panel and EUCAST E.DEF 9.3.2, respectively)	Prophylactic coverage with FLC FLC and L-AMB (7 days)	Death (No clearance of BC after 7 days of treatment with FLC and L-AMB)

*M* Male, *F* Female, *ALL* Acute lymphoblastic leukaemia, *AML* Acute myeloid leukaemia, *HIV* Human immunodeficiency virus, *HSCT* Hematopoietic stem-cell transplant, *GVHD* Graft-versus-host disease, *UCBT* Umbilical cord blood transplantation, *COPD* Chronic obstructive pulmonary disease, *CVC* Central venous catheter, *PICC* Peripherally inserted central catheter, *CVAD* Central venous access device, *ELBW* Extremely low birth weight, *RDS* Respiratory distress syndrome, *NEC* Necrotizing enterocolitis, *BC* Blood culture, *AMB* Amphotericin B, *5-FC* Flucytosine, *ITC* Itraconazole, *FLC* Fluconazole, *MFG* Micafungin, *VRC* Voriconazole, *L-AMB* Liposomal amphotericin B, *AFG* Anidulafungin, *POS* Posaconazole, *CAS* Caspofungin, *TRB* Terbinafine, *MCZ* Miconazole, *ISA* Isavuconazole, *ND* Not determined, *NA* Not available

An overview of the literature indicated that echinocandins proved to be ineffective against *E. dermatitidis* infections [23]. Only the combinations of CAS with VRC, AMB or ITC presented synergic activity against this fungus [24]. Although monotherapy with an echinocandin does not seem to be effective, the AFG MIC in case 10 (CLSI M38-A2) [10] was notably low (0.008 mg/L) and as the patient did not have evidence of deep organ seated infection, the therapy was not changed. However, he eventually died due to haemoptysis and supraventricular tachycardia. As echinocandins have not good activity against *E. dermatitidis*, a prophylactic administration of MFG [25] may cause fungal predominance and help manifestation of infection. Besides this, diagnosis and selection of the appropriate treatment through susceptibility testing is difficult due to the pathogen’s rarity and slow growth. Most *in vitro* active antifungal agents have been found to be VRC and terbinafine (TRB), followed by POS, ITC and AMB in rank order of decreasing activity on mg/L basis [26]. The azoles have been the primary agents because of excellent *in vitro* activity, safety in long-term use and clinical experience. Polyene drugs show good antifungal activity *in vitro* and have been used successfully in some cases of disseminated disease. FLC and 5-FC have variable activity and in particular 5-FC should always be used in combination with another agent due to rapid development of drug resistance [7]. However it is important to note that there are no clinical breakpoints or clinical trials available to evaluate efficacy of antifungal agents in this group of fungi [7]. *E. dermatitidis* has unique difficulties when preparing an inoculum for susceptibility testing (both the yeast and mould phase are present within the same colony resulting in a mixed population of fungal forms), while the ideal reading time is unknown due to their low growth rate [27]. Furthermore, there are no established MIC breakpoints to interpret susceptibility results in a standardized fashion, whereas susceptibility data are limited showing a very broad MIC interval, particularly for echinocandins (e.g. TRB 0.06–0.5 mg/L, AMB 0.01–1 mg/L, ITC ≤ 0.03–0.5 mg/L, VRC 0.06–1 mg/L, POS 0.016–0.25 mg/L, ISA 0.03–1 mg/L, FLC 2–32 mg/L, echinocandins 0.008- > 16 mg/L, 5-FC 2- > 64 mg/L) [18, 27, 28].

Regarding echinocandin therapy, death was occurred when echinocandins were given as treatment (cases 8 and 10) or prophylaxis (cases 7 and 9). In particular, case 7 concerned a patient on MFG prophylaxis who had multiple and severe risk factors, such as hematopoietic stem-cell transplantation (HSCT) and GVHD. Death was provoked 10 days after discontinuation of antifungal combination therapy of liposomal AMB with VRC due to transition to comfort care measure [18]. Besides that, it is known that *E*. *dermatitidis* must be considered as a possible cause of opportunistic invasive mycosis especially in patients with long immunosuppression after allogeneic HSCT with chronic GVHD [18]. In case 9, the patient developed *Exophiala* infection while on MFG prophylaxis and died despite of CVC removal and liposomal AMB therapy for 49 days, as he suffered by hematologic malignancy (acute myeloid leukaemia), myelofibrosis and prolonged neutropenia [20]. Furthermore, in case 11, blood culture remained positive despite empiric therapy with MFG for 12 days and sterilised only when liposomal AMB was added to antifungal therapy [21]. The length of therapy in the diseases caused by phaeoid fungi generally ranges from several weeks to months or longer [25]. As shown in Table 1, prolonged antifungal therapy was necessary in most of the survived patients. Also, in all cases, except the present, CVC removal was combined with antifungal therapy.

Of note, an early and accurate diagnosis is of great importance for a possible good outcome and underlines the need for identification methods that may lead to an appropriate antifungal treatment. Nevertheless diagnosis to the species level is challenging due to *Exophiala’s* similar synanamorphs produced during its complex life cycles. In addition, susceptibility testing results are not promptly available [29]. The infection is often misdiagnosed because these pathogens are not included in the currently available commercial databases. On the other hand, molecular methods based on DNA sequence analysis as well as matrix-assisted laser desorption ionization time-of-flight mass spectrometry have demonstrated accurate results in the identification of black yeasts to the species level [29].

Our neonate had severe risk factors, such as prematurity, ELBW, RDS, intubation, mechanical ventilation, CVC, NEC, parenteral nutrition, abdominal surgery and use of antibiotics. It is difficult to prove the source of infection since there was not any special surveillance in the NICU. Evidence regarding transmission routes in the hospital setting is scarce, but outbreaks due to direct iatrogenic inoculation (fungus-contaminated medication) have been reported. A nosocomial outbreak of *E. jeanselmei* fungemia related to contaminated deionized water from the pharmacy has been described [30]. An outbreak of *E. dermatitidis* meningitis or arthritis related to epidural or intra-articular injections of contaminated methylprednisolone acetate has been recorded [31]. In an outbreak at an oncology clinic, despite the fact that all patients were exposed to a contaminated intravenous solution, only those with CVC developed infection [32]. In the present case no surveillance of the preparation and administration of intravenous medication was made neither ΝICU surfaces were cultured in search for fungi. Nevertheless, no other neonate presented *E. dermatitis* fungemia later on. We consider that the fungus could have probably entered bloodstream during the intravenous infusion. The Hickmann CVC that was not removed may have favoured adherence, biofilm formation and persistence of fungemia. It has been shown that 92% of clinical isolates of *E. dermatitidis* exhibit biofilm formation and for that reason should be always considered as a probable agent that cause CVC-associated fungemia [10].

Regarding the seven cases that patients survived, azoles were proved to be the most effective antifungal: firstly ITC (cases 2, 3, 5), followed by FLC (cases 4, 12) and VRC (case 11). However, in case 6 empric therapy with fluconazole failed against an isolate with MIC 48 mg/L and in our case fluconazole prophylaxis failed against an isolate with MIC 8 mg/L (50% growth inhibition) and 16 mg/L (>90% growth inhibition) with the EUCAST reference method indicating resistance based on *Candida* non-species specific breakpoints for fluconazole (susceptible ⩽2 mg/L, resistance >4 mg/L). Moreover, the prolonged antifungal therapy was necessary for up to 5 months (case 3). Only two patients who survived had received combination therapy (AMB and 5-FC in case 1 and MFG and liposomal AMB/VRC in case 11). All seven patients had a CVC removal in combination with antifungals. Echinocandins should be avoided as they were administered as therapy in 3 cases (in cases 8 and 10 with fatal outcome and in case 11 with failure). Only one patient of those who survived had received prophylactic antifungal therapy (case 11) (the remaining 4 patients with prophylactic treatment died). Prophylactic strategies are important in immunosuppression but may cause fungal predominance, treatment failure and fatal outcome without CVC removal.

In conclusion, it is widely accepted that better prognosis of the patients is associated with the early diagnosis and timely treatment. However, in our case, a major contributing factor to unfavourable prognosis was prematurity and its associated comorbidities. Moreover, due to the rarity of *E. dermatitidis* infections, they are usually underestimated and little is also known about the characteristics of this rare yeast and the optimal antifungal therapy. Furthermore**,** current antifungal agents that are available for treatment of neonatal fungemia are based on guidelines for adults. CVC should be removed and antifungal therapy with an azole (ITC or FLC after susceptibility testing) or AMB monotherapy should be administered for at least 3 weeks until blood culture sterilisation and clinical improvement. Echinocandins and AMB plus azole combination therapy should be avoided. Further studies and continuous surveillance are warranted in order to plan efficacious prophylactic and therapeutic strategies.

## Data Availability

All data generated or analysed during this study are included in this published article.

## Abbreviations

M: Male
F: Female
ALL: Acute lymphoblastic leukaemia
AML: Acute myeloid leukaemia
HIV: Human immunodeficiency virus
HSCT: Hematopoietic stem-cell transplant
GVHD: Graft-versus-host disease
UCBT: Umbilical cord blood transplantation
COPD: Chronic obstructive pulmonary disease
CVC: Central venous catheter
PICC: Peripherally inserted central catheter
CVAD: Central venous access device
ELBW: Extremely low birth weight
RDS: Respiratory distress syndrome
NEC: Necrotizing enterocolitis
AMB: Amphotericin B
5-FC: Flucytosine
ITC: Itraconazole
FLC: Fluconazole
MFG: Micafungin
VRC: Voriconazole
L-AMB: Liposomal amphotericin B
AFG: Anidulafungin
POS: Posaconazole
CAS: Caspofungin
TRB: Terbinafine
MCZ: Miconazole
ISA: Isavuconazole
ICU: Intensive care unit
NICU: Neonatal intensive care unit
CRP: C-reactive protein
IFIs: Invasive fungal infections
WBC: White blood cells
PLTs: Platelets
MIC: Minimum inhibitory concentration
NCPAP: Nasal continuous positive airway pressure
PEEP: Positive end-expiratory pressure
spp: Species
ND: Not determined
NA: Not available
